# Bacterial Cellulose (BC) and BC Composites: Production and Properties

**DOI:** 10.3390/nano12020192

**Published:** 2022-01-07

**Authors:** Tatiana G. Volova, Svetlana V. Prudnikova, Evgeniy G. Kiselev, Ivan V. Nemtsev, Alexander D. Vasiliev, Andrey P. Kuzmin, Ekaterina I. Shishatskaya

**Affiliations:** 1 School of Fundamental Biology and Biotechnology, Siberian Federal University, 79 Svobodny Pr., 660041 Krasnoyarsk, Russia; sprudnikova@sfu-kras.ru (S.V.P.); evgeniygek@gmail.com (E.G.K.); ivan_nemtsev@mail.ru (I.V.N.); adva@iph.krasn.ru (A.D.V.); shishatskaya@inbox.ru (E.I.S.); 2 Institute of Biophysics SB RAS, Federal Research Center “Krasnoyarsk Science Center SB RAS”, 50/50 Akademgorodok, 660036 Krasnoyarsk, Russia; 3 L.V. Kirensky Institute of Physics SB RAS, Federal Research Center “Krasnoyarsk Science Center SB RAS”, 50/38 Akademgorodok, 660036 Krasnoyarsk, Russia; 4 Federal Research Center “Krasnoyarsk Science Center of the Siberian Branch of the Russian Academy of Sciences”, 50 Akademgorodok, 660036 Krasnoyarsk, Russia; 5 School of Petroleum and Gas Engineering, Siberian Federal University, 79 Svobodny Pr., 660041 Krasnoyarsk, Russia; akuzmin@sfu-kras.ru

**Keywords:** bacterial cellulose, composites, production, properties

## Abstract

The synthesis of bacterial cellulose (BC) by *Komagataeibacter xylinus* strain B-12068 was investigated on various C-substrates, under submerged conditions with stirring and in static surface cultures. We implemented the synthesis of BC on glycerol, glucose, beet molasses, sprat oil, and a mixture of glucose with sunflower oil. The most productive process was obtained during the production of inoculum in submerged culture and subsequent growth of large BC films (up to 0.2 m^2^ and more) in a static surface culture. The highest productivity of the BC synthesis process was obtained with the growth of bacteria on molasses and glycerol, 1.20 and 1.45 g/L per day, respectively. We obtained BC composites with silver nanoparticles (BC/AgNPs) and antibacterial drugs (chlorhexidine, baneocin, cefotaxime, and doripenem), and investigated the structure, physicochemical, and mechanical properties of composites. The disc-diffusion method showed pronounced antibacterial activity of BC composites against E. coli ATCC 25922 and S. aureus ATCC 25923.

## 1. Introduction

Cellulose is the most abundant biopolymer on Earth, being the main structural component of the plant cell wall [1]. Cellulose is also synthesized by some ocean animals (tunicates), in which case it is called tunicin [2]. A significant source of cellulose is microbiological synthesis using algae, fungi [3], and bacteria of various taxa, -*Agrobacterium, Sarcina, Rhizobium*, and *Gluconacetobacter* (formerly *Acetobacter*) [4,5,6]. The species *Gluconacetobacter xylinus*, currently classified as *Komagataeibacter medellinensis*, is the most actively studied producer of bacterial cellulose due to the highest production characteristics and the ability to use a variety of compounds as a carbon source [7,8]. Bacterial cellulose (BC) is in demand for various purposes due to its advantages over plant cellulose: high purity; absence of impurities (hemicellulose, lignin, etc.); the possibility of synthesis from various substrates, including C-containing industrial waste; high rates of growth and productivity of producer strains [9]. Structurally, BC belongs to the type of crystalline nanofibrillated cellulose, with fiber sizes ranging from 5 to 100 nm. BC exerts high water holding capacity and unique mechanical properties precisely because of the nanofibrillar 3D structure of BC-film, consisting of randomly oriented nanofibers [10,11]. 

Cellulose is used in a wide spectrum of applications in food and paper industries, medicine, and pharmaceutics [11,12,13]. Currently, extensive data has been accumulated on various bacterial strains capable of synthesizing BC, which has different physicochemical properties that make BC promising material for biological applications [14,15,16]. It was found that the type of producer and fermentation conditions could have a significant effect on BC properties, including the packing of fibrils, density, porosity, and mechanical strength of BC films. It has been shown, for example, that the genetically engineered strains of *G. xylinus*, with genes from *Candida albicans*, are capable of producing BC with improved in vivo biodegradability. The cellulose synthase of *G. xylinus* can utilize both UDP-glucose and UDP-N-acetylglucosamine (UDP-NAcG) as substrates [16,17]. The presence of NAcG makes BC susceptible to the lysozyme and also disrupts the highly-ordered crystalline structure of BC, which makes cellulose in a lesser degree rigid and crystalline [18].

The BC properties are significantly different from those of plant cellulose, making it possible to use BC in various fields, from physics and chemistry to medicine and mechanical engineering. BC has a highly porous structure with high permeability to liquids, favorable for cell adhesion and proliferation, as well as a high ability to absorb water, up to 90%. These properties of BC are due to its ultrafine reticular structure, consisting of ribbon micro and nanofibrils, which are 100 times thinner than plant cellulose fibers [19]. BC possesses exceptional mechanical characteristics with deformability resembling soft tissue [19,20]. The breaking stress of BC films can be 2 × 106 Pa, despite the 99% water content. BC also has exceptional mechanical properties in dry conditions due to its crystalline nanofibrillar network. The tensile strength of one BC fiber is almost comparable to Kevlar^®^ and steel [21], which makes BC promising for applications in the fields where high mechanical characteristics are required. 

BC is a non-allergenic biopolymer [22]. The structure and mechanical properties of BC make this biomaterial an ideal candidate for tissue engineering scaffolds [23], including artificial cartilage [20,24], heart valve prostheses [25], artificial blood vessels [26,27], corneal sheath [28], for dental root canal treatment [29], and skin tissue repair [30], as a structuring agent in cleaning formulations [31]. BC is used in cosmetology as a natural facial scrub [32] and face masks [33].

The degradation product of BC is glucose. This fact explains the high biocompatibility of BC and makes it a promising material for many applications, primarily for reconstructive surgery [34]. The BC is approved by the FDA (USA) for use by Xylos Corp. (Langhorne, PA, USA) as MTA^TM^ Surgical Sheet, Xylos^®^ Vessels, Xylos^®^ Porous Surgical Mesh, Securian^TM^ Tissue Reinforcement Matrix [13]. BC exhibited higher complement activation parameters compared to conventional materials such as polyethylene terephthalate (PET) and expanded polytetrafluoroethylene (ePTFE) [35]. A study of thrombogenicity in human blood plasma showed that BC induces slower coagulation than clinically available materials such as Dacron^®^ and Gore-Tex^®^. BC and its composites have good hemocompatibility with low factor XII and platelet activation compared to PTFE [36].

To impart additional properties to BC, such as antibacterial activity, improvement of cell adhesion and proliferation, BC-based composites with various materials are designed [37,38]. Composites of chitosan [14], collagen [39], sodium alginate, gelatin, and polyethylene glycol have been described [37]. BC alone does not have any antibacterial activity, but in combination with chitosan, alginate, nanosilver, etc., it can inhibit the growth of pathogenic microorganisms *E. coli*, *C. albicans*, and *Staphylococcus aureus* [14,40,41,42]. Available biomedical and pharmaceutical applications of BC-based composites include antibacterial and antiviral films and systems for the healing of skin wounds, cardiovascular system, skeletal muscle, and cartilage, as well as drug delivery systems [43].

BC’s great prospects are associated with a modern innovative direction focused on developing highly functional nanocrystalline materials based on cellulose [44]. The created bionanomaterials have a large number of hydroxyl groups, a large specific surface area, high biocompatibility, mechanical strength, relatively low cost, and great biodegradability potential. Therefore, they are currently considered to be promising candidates for various applications, especially in biomedical fields. Bacterial nanocellulose, in addition to classical medicine [45,46], tissue engineering [47], and drug delivery [48,49], has great potential for cosmetology [50], food [51], textile and footwear industries [52], and other applications [53]. However, the use of BC depends on the economic feasibility of its production processes. Although BC is in great demand in various fields, its production is still a costly process. Only the cost of synthetic nutrient media used for the microbiological synthesis of BC can be up to 65% of the total cost of the process [54]. Therefore, the expansion of the scale and scope of bacterial cellulose depends on the availability of productive strains that provide high yields of this valuable biotechnological product with the usability of more accessible substrates. This actualizes research aimed at the development and implementation of effective technologies for BC biosynthesis.

This work presents the results of a study of BC synthesis by the strain of acetic acid bacteria *Komagataeibacter xylinus* B-12068 on various C-substrates, including waste; also, the production and characteristics of BC composites with nanosilver and antibacterials were evaluated.

## 2. Materials and Methods

### 2.1. Production of Bacterial Cellulose

Bacterial cellulose was synthesized by *Komagataeibacter xylinus* strain B-12068 isolated from the fermented tea (kombucha) *Medusomyces gisevii* J. Lindau on Hestrin-Schramm (HS) medium [55]. The strain was deposited in the Russian National Collection of Industrial Microorganisms (VKPM) (Accession No. B-12068). The phenotypic and cultural properties of the strain were described in detail else were [56,57].

Fermentation was conducted in 0.5 L flasks containing 100 mL of the medium and in shaker incubator Innova 44 (Eppendorf, Framingham, MA, USA) at constant temperature 30 °C and 200 rpm. To scale up the process, we used an automated fermentation complex Bio-Flo 115 with a fermenter volume of 8 L containing 4 L of the medium. Surface liquid-phase cultivation of bacteria was carried out in stainless-steel trays with 0.4 × 0.5 × 0.03 m; the volume of the culture medium was 1 L.

BC biosynthesis was studied on the standard HS medium modified by changing C-substrates: glucose (dextrose), analytical grade (Khimreactivsnab, Ufa, Russia), purified glycerol, 99.3% (Corporate Oleon, Sandefjord, Norway), refined sunflower oil (Zolotaya Semechka, Rostov-on-Don, Russia), beet molasses (LLC “Ertilsky sugar”, Moscow, Russia), and sprat oil. Sunflower oil predominantly contained linoleic (18:2ω6), oleic (18:1ω9), and palmitic acids (16:0) in the amount of 63.1, 24.5, and 7.4% of the total FA, respectively; the saturation index was 0.14. The complete composition is described in Volova et al. [58].

The original beet molasses had the following composition (% by weight): dry matter 79.6; sucrose 46.8; total nitrogen 1.5–2; betaine 4–7; conductometric ash 6–11. The sprat oil is a waste product from the Baltic sprat (*Sprattus sprattus*) meal production, obtained by extracting fat from heads at a temperature of 60–90 °C. Sprat oil contains 95% lipids, 4% proteins, and 1% carbohydrates. The lipids contain 16 fatty acids (FA); the dominant FAs were palmitic (28%), oleic (25.3%), docosahexaenoic (16.7%), and timnodonic (8.7%) acids.

The synthesized BC was separated from the culture liquid, purified in a 0.5% NaOH solution for 24 h at 25–27 °C, neutralized in a 0.5% solution of HCl for 24 h, and afterwards rinsed it in distilled water until pH 7. The BC pellicles were stored in sterile normal saline or air-dried at 45 °C for three days until they reached a stable weight. The dried BC pellicles were weighed on the analytical balance (Ohaus, Greifensee, Switzerland).

The total BC yield and biosynthesis productivity for different fermentation processes were evaluated using conventional methods. The BC yield (X) was calculated as X = g/L or the weight of dry cellulose (g) per liter of the medium. The BC productivity (P) was calculated as P = g/L·day^−1^.

### 2.2. Production of Bacterial Cellulose Composites

BC composites with silver nanoparticles (BC/AgNPs) were produced by a hydrothermal method [59]. Purified raw BC films were placed in flasks with 0.01, 0.05, and 0.10 g/L of AgNO_3_, and heated for 60 min at a temperature of 90 °C. Composite BC films were lyophilized at a temperature of −40 °C and pressure of 0.12 mbar for 24 h in a vacuum drying unit ALPHA 1-2/LD (Martin Christ Drying Systems GmbH, Osterode, Germany) or kept at room temperature in a laminar flow cabinet for 24 h. The parameters of the produced silver nanoparticles were investigated with a Zetasizer Nano ZS particle analyzer (Malvern, UK), using dynamic light scattering, electrophoresis, and laser Doppler anemometry. 

In order to impart bactericidal properties to cellulose, BC composites with antibacterial drugs (chlorhexidine, baneocin, cefotaxime, doripenem) were prepared. The dried BC films were immersed in 0.05% water solution of drugs for 24 h, then removed from the solutions, washed in distilled water, and dried at room temperature. After that, 7 mm diameter discs were cut from the BC films.

### 2.3. A Study of Microstructure, Physicochemical, and Mechanical Properties of Bacterial Cellulose Composites

The microstructure of the surface of the BC and BC composites was analyzed using scanning electron microscopy (S-5500, Hitachi, Tokyo, Japan). Prior to the analysis, the pellicles were freeze-dried in an ALPHA 1-2/LD freeze dryer for 24 h. 5 × 5 mm samples were placed onto the sample stage and sputter-coated with platinum, using an Emitech K575XD sputter coater (10 mA, 2 × 40 s). Fiber diameters were measured by analyzing SEM images using the ImageJ program. The diameters of 50 individual ultrafine fibers were estimated in 10 fields of SEM in triplicate. 

The chemical composition of BC/AgNPs was analyzed by determining C/O/N using a Flash EA 1112 CN analyzer (Thermo Fisher Scientific Inc., Waltham, MA, USA). The analysis for major and trace elements was performed with an iCAP 6000 inductively coupled plasma emission spectrometer (Thermo Fisher Scientific Inc., Waltham, MA, USA) after wet mineralization of cellulose samples with a mixture of perchloric and nitric acids.

BC samples were analyzed using FTIR spectroscopy. IR spectral data were collected on the 400–4000 cm^−1^ range using a NICOLET 6700 FTIR spectrometer (Thermo Scientific, Waltham, MA, USA) and a Smart Orbit accessory by the attenuated total reflection (ATR) technique.

Thermal analysis of BC and BC composites was performed using a TGA (Mettler-Toledo AG, Greifensee, Switzerland). A weighed portion of samples from 2 to 5 mg was heated from 50 to 500 °C at a rate of 100 °C/min. The onset of sample weight loss (Tdegr) was recorded on the thermograms analyzed using the STARe v11.0 software. 

X-ray structure analysis and crystallinity determination were performed using a D8 ADVANCE X-ray powder diffractometer (Bruker AXS GmbH, Hamburg, Germany), CuKa radiation. In order to determine the crystallinity (C_x_) of BC, the data were collected using a Vantec high-speed detector, with an exposure time of 3000 s. X-ray tube operating mode was 40 kV and 40 mA. Mechanical properties of the wet BC composites were investigated using an Instron 5565 electromechanical tensile testing machine (U.K.). The samples were maintained at ambient temperature in a laminar cabinet for at least two weeks for reaching equilibrium crystallization. At least five samples were tested for each type of pellicle. Measurements were conducted at ambient temperature; the clamping length of the samples was 30 mm. The speed of the crosshead was 3 mm/min at ambient temperature. Young’s modulus (E, MPa) and elongation at break (ε, %) were automatically calculated by the Instron software (Bluehill 2, Elancourt, France). The software calculated the slope of each stress–strain curve in its elastic deformation region for obtaining Young’s modulus. Measurement error did not exceed 10%.

### 2.4. In Vitro Antibacterial Tests

The direct inhibitory effect of BC/AgNP and BC/antibiotic composites was tested on cultures of reference strains—*Escherichia coli* ATCC 25922 and *Staphylococcus aureus* ATCC 25923, using disk diffusion method on Petri dishes with Mueller-Hinton (MH) agar. The 0.5 McFarland standard suspensions of bacterial isolates (approximately 1.5 × 10^8^ CFU/mL) were inoculated on a sterile solid medium. The composite discs of BC with AgNP or antibiotics were placed on the surface of the inoculated medium (three discs per plate). The Petri dishes were incubated at a temperature of 37 °C for 24 h. The diameter of the growth inhibition zones was measured by photographs of dishes using the ImageJ program. The means and standard deviations were calculated. 

### 2.5. Statistical Analysis

Statistical analysis was performed using Microsoft Excel 2010 and the Statistica 6.0. Comparison of two related groups for quantitative attributes was performed using the parametric method with the Wilcoxon matched-pairs test. Comparison of two independent groups for quantitative attributes was performed using the nonparametric method with the Mann–Whitney *U* test. Differences were considered statistically significant at a *p*-value of 0.05.

## 3. Results and Discussion

Physiological and biochemical characteristics of producer strains determine the choice of carbon substrate for BC biosynthesis, among which hexoses, pentoses, ethanol, organic acids, and glycerol, are described [60,61,62,63]. The BC yield and its structure depend on the specifics of the producer and the cultivation modes (static surface mode or submerged culture with stirring, the ratio of the volume and area of the medium, the pH value, the presence of additives in the nutrient medium—ethanol, acetate, starch, hemicellulose, etc.) [30,62,64,65,66]. In general, the published results on the synthesis of BC indicate the need to optimize the biosynthesis modes and increase the production characteristics of strains of acetic acid bacteria, as well as the involvement of various, including new and more accessible substrates.

### 3.1. Production Characteristics of the K. xylinus B-12068 Strain Grown on Various C-Substrates 

Using our strain of acetic acid bacteria *K. xylinus* B-12068 to synthesize BC, we have previously investigated and showed the effect of the type of sugar on cellulose production. During seven days of cultivation in glass flasks at 30 °C and initial pH 6.0, we obtained the highest BC yield on the standard HS medium with glucose (2.2 g/L), a little less on the HS medium with sucrose (1.6 g/L) and galactose (1.4 g/L). Maltose and mannitol did not support the growth of this bacterial strain [57].

The key concern of BC biotechnology is increasing the production parameters of the biosynthesis process and reducing the cost of carbon raw materials to increase the availability of this valuable product. The first goal was to investigate the possibility of attracting several new C-substrates, including unusual ones, for BC synthesis. This work presents the results of cultivating the strain B-12068 on various carbon sources, including waste, compared to glucose and cultivating in various modes (surface and submerged fermentation, in static and stirring modes). As a C-substrate, sugar beet molasses, sprat oil, glycerol, and a mixture of glucose with sunflower oil (ratio 3:1) were used. The concentration of the C-substrate in the medium was 21 ± 1 g/L. 

**Figure 1:**
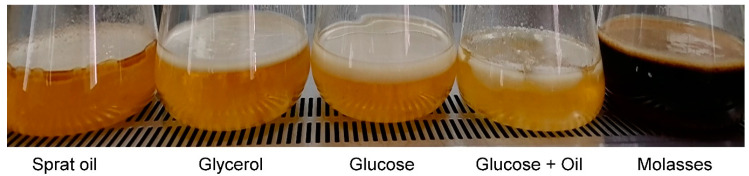
Formation of BC films in culture of *K. xylinus* B-12068 on various C-substrates.

At the first stage, the cultivation of bacteria was carried out in 0.5 L glass flasks on 0.1 L of the stirred nutrient medium at 200 rpm and 30 °C. All substrates supported bacterial growth and BC synthesis. In all variations, the formation of stable films on the culture medium surface was noted on the third day (Figure 1, Table 1).

The highest BC yield was obtained on the HS medium with glycerol—3.90 ± 0.30 g/L. On the HS medium with beet molasses (sugar concentration in the medium was 20–25 g/L), the BC yield reached 3.2–3.3 g/L, comparable to the BC yield when *K. xylinus* was cultured on a standard HS medium with glucose. An increase in the concentration of molasses up to 40 g/L per sugar led to the suppression of BC synthesis but did not stop it completely. During the cultivation of bacteria on twofold diluted molasses (sugar concentration 200–220 g/L), without adding the main components of the medium, such as peptone, yeast extract, phosphorus, nitrogen, the bacterial film became thin and wrinkled. The inhibition of BC synthesis by high concentrations of molasses was also noted in other studies [67]. The smallest in five days BC production was observed on a medium containing sunflower oil and glucose—2.21 ± 0.49 g/L. It may be due to the stratification of the water-oil suspension and substrate competition. Nutrient media containing lipid substrates were mechanically dispersed to obtain stable emulsions, after which the inoculum was added. However, by the time the cellulose film was formed, the emulsion was significantly destroyed, which led to the formation of an oxygen-impermeable layer and a decrease in the availability of the substrate.

Thus, we showed the possibility of various C-substrates attraction for the synthesis of BC, including unusual substrates or wastes. The next task was aimed at increasing the productivity of BC biosynthesis.

One of the most productive fermentation methods in biotechnology that maximizes product yield is submerged cultivation. Some works have shown that dynamic conditions contribute to the synthesis of BC.

For increasing the productivity of BC synthesis, we studied the method of submerged cultivation in a fermentation apparatus. The bacteria were cultured in an 8 L Bio Flo 115 fermenter with a fill factor of 0.5. To test this regime, we used glucose as a carbon source. As in the process of deep fermentation, it becomes necessary to provide an unlimited supply of nutrients and oxygen to producing cells, the most important thing is to ensure optimal conditions for mass transfer throughout the entire volume of the nutrient medium. In this regard, we studied the influence of the intensity of mass transfer in the fermenter on the output of the BC and the productivity of this process, including the rotation speed of the mixer and the amount of air supplied (Table 2).

As follows from the data, the highest BC yield (3.33 g/L) and productivity (0.48 g/L·day^−1^) were obtained with the stirring mode 500 rpm and the amount of supply air of 0.4 L/min per L of the medium. A decrease in the value of X (g/L) comes amid an increase in the stirrer speed. It results from the high turbulence of the liquid phase in the fermentation vessel and the death of the part of cells. The decrease in rotation speeds did not have a positive effect and worsened productivity. Increasing the aeration of the culture in the fermenter also had a negative impact. The results obtained are consistent with the data of Jung et al. [68]. The authors studied the effect of the stirrer revolutions in the fermenter on the mutational variability of the bacterial population, BC producer. With a decrease in the number of revolutions, there was an increase in the population of Cel^−^ mutants that lost the ability to synthesize BC and switched to the synthesis of glucuronic acid. In another work, the authors showed that increased oxygen stress in the medium could be accompanied by the formation of glucuronic acid and a decrease in the production of BC [69].

Thus, the conducted submerged cultivation of *K. xylinus* B-12068 in a fermenter with a change in the mixing mode and oxygen transport did not increase the productivity of the BC synthesis by the studied strain. In addition, the bulk of the excreted cellulose is deposited on the internal structure of the fermenter. It complicates the operation of the sensors and also leads to a change in the hydrodynamic characteristics of the fermenter and, consequently, to a deterioration in mass transfer. Another disadvantage of submerged culture is that BC does not form a film, as it does in static conditions. It includes granules and particles suspended in the medium. 

The positive result of studying the process in the fermenter was the discovered effect of a more active increase in the biomass of bacteria in submerged culture with stirring compared to a static method without stirring (for example, in Petri dishes or glass and metal trays). So, per day, the number of acetic acid bacteria in the fermenter increased from 0.54 × 10^8^ to 1.2 × 10^8^ cells per mL of culture; that is, it doubled. In a static surface culture, the doubling time of the biomass of acetic acid bacteria lasts from 3 to 4 days.

We used the discovered effect of the increased bacterial growth rate in the fermenter to develop the original two-stage process. It includes promptly obtaining active inoculum in the fermenter at the first stage and the subsequent growth of cells and BC film formation in a static surface mode at the second one.

To work out the mode ensuring the production of active inoculum of *K. xylinus* B-12068 with a high starting concentration of bacterial cells, we carried out the cultivation process with pH regulation. It is known, the pH value affects the growth of acetic acid bacteria. According to Yassine et al. [70], pH 5.0 leads to a high level of BC formation and an increase in BC producers and provides a high activity of the cellulose synthase enzyme [71]. The active reaction of the culture medium in the fermenter was stabilized on the pH level 5.0–5.5 by titrating the culture medium with 0.1 N KOH solution. Varying the process’s mode and duration provides obtaining an inoculum with a high bacterial concentration—6.4 × 10^8^ cells per mL. It is crucial since the use of inoculates with a low initial cell concentration in biotechnological processes is accompanied by a lengthening of the lag phase and an increase in the total duration of the process.

At the next stage, the synthesis of BC was investigated during the static surface liquid-phase fermentation, which provides the formation of particularly valuable forms of bacterial cellulose shaped as films, which have excellent prospects for use in reconstructive surgery and cell and tissue engineering technologies [20,23,24,25,26,27,28,29,30].

It should be noted that the process of BC biosynthesis is aerobic, and cellulose synthesis is carried out by cells located at the air–water interface. There is a suggestion that the BC film helps bacteria float at the air–liquid interface, which optimizes access to oxygen [72]. When carrying out the fermentation process under the static surface mode, increasing the air–water contact area (the ratio of area to volume—S/V) becomes a key factor. When implementing the process of growing bacteria *K. xylinus* B-12068 in stainless trays with an area of S = 0.2 m^2^, the contact of the S/V phases was 0.2. It is much higher than the indicator in 0.5 L flasks (S/V = 0.07). The increase in the indicator was accompanied by an increase in the productivity of the BC synthesis by two or more times. We studied the static surface liquid-phase process of bacterial cultivation in two modes. The first mode was a one-stage process. The cultivation and production of BCs were carried out in fermentation trays in 1 L of a nutrient medium inoculated with a bacterial suspension obtained from a museum culture (the volume of inoculum was 10%). The cultivation was conducted using five different C-substrates, similar to those previously described (Figure 1, Table 1). The second mode was a two-stage process: the inoculum was grown for three days in a fermenter with stirring at the first stage, and BC was produced in fermentation trays at the second stage. For this, 10 to 100% inoculum was added to 900 mL of HS medium containing 20–25 g/L of C-substrate. One liter of the resulting suspension was poured into a stainless-steel tray, the thickness of the culture medium layer was 8–10 mm. The cultivation was carried out for 5 days at 30 °C. After that, obtained cellulose was filtered on a Buchner funnel and washed with a KOH solution.

**Figure 2:**
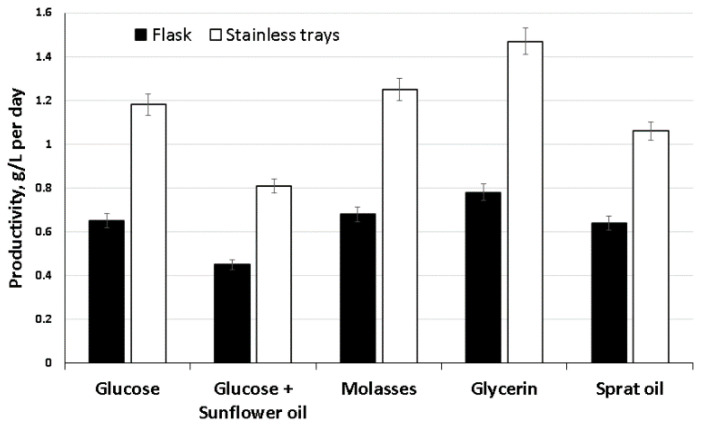
Biosynthesis of BC films by the strain *K. xylinus* B-12068 on different ratios of the area to volume (S/V) in glass flasks (S/V = 0.07) and trays (S/V = 0.2).

A comparative study of one-stage surface cultivation of bacteria in trays and glass flasks is shown in Figure 2. As follows from the data, the productivity of the BC biosynthesis process depended on the interface area. It was significantly higher (50–100%) for all substrates when the process was carried out in trays compared to the results on the same substrates in a flask. We obtained the highest productivity of BC for five days of cultivation of acetic acid bacteria in trays on glycerol—yield 7.35 g/L (1.47 g/L∙day^−1^). It surpassed the results of the Korean researchers, who obtained a BC yield of 4.98 g/L in seven days [73]. The minimum productivity was obtained on a mixed substrate—glucose with sunflower oil (0.81 g/L∙day^−1^). Average productivity indicators were obtained by cultivation on sprat oil and molasses (1.06 and 1.25 g/L∙day^−1^, respectively). The use of molasses as a C-substrate provided better BC production than glucose (1.18 g/L∙day^−1^). In some works [74,75], it is also noted that molasses is a promising substrate that provides higher BC production than glucose.

**Figure 3:**
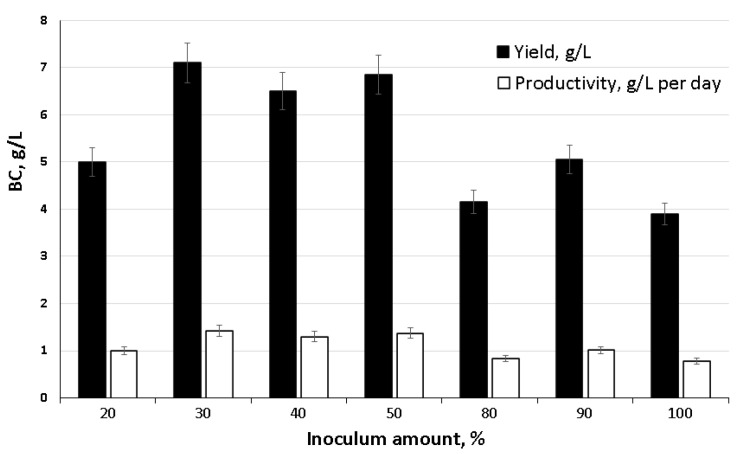
Influence of the amount of introduced inoculum on the biosynthesis of BC by the strain *K. xylinus* B-12068.

The results of the second two-stage surface–liquid-phase synthesis of BC with preliminary inoculum preparation in a fermenter are shown in Figure 3. It was found that the volume of inoculum introduced into the tray influenced the yield of BC and the productivity of the biosynthesis process.

We noted the active release of cellulose into the medium and the formation of a well-formed BC film on the surface on the second day of introducing 30% or more of the inoculum from the total amount of the starting medium. The highest indicators of BC yield (6.5–7.1 g/L) and productivity (1.3–1.42 g/L∙day^−1^) were obtained using the volume of inoculum 30 up to 50%. Less or more inoculum negatively affected production characteristics.

The costliest part of the BC synthesis process is the components of the medium, primarily the C-substrate and the growth-stimulating compounds that are not entirely utilized by the culture in one cycle of bacterial cultivation. The surface culture method, in which obtained BC film is collected from the surface, and a new portion of inoculum is introduced into the medium, allows shortening the second fermentation cycle since a certain number of bacterial cells are left after the first fermentation. Moreover, an additional portion of BC can be obtained with fuller use of the culture medium components. When we carried out the fermentation mode with a double collection of BC films, it allowed increasing BC yield up to 14 g/L, which twice surpassed the yield in the single inoculation mode.

**Figure 4:**
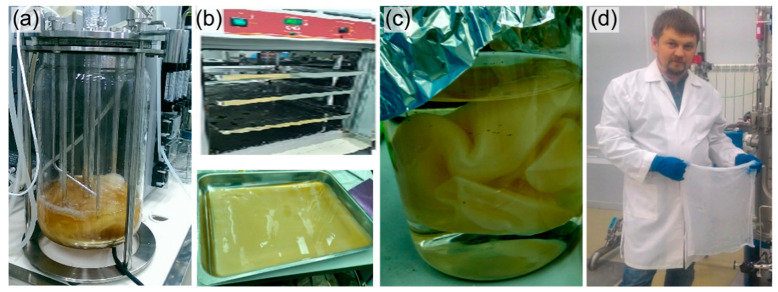
The two-stage process of BC production: (**a**) production of inoculum in submerged culture with stirring; (**b**) BC synthesis in surface liquid-phase mode; (**c**) cleaning of BC films from bacteria and medium components; (**d**) finished product.

This study has provided the implementation of various modes of BC synthesis on different substrates, including waste. It is significant that this mode and the selected carbon sources not only offered high production indicators of the culture but also implemented the production of large BC films (up to 0.2 m^2^ and more) (Figure 4).

Comparison of the obtained results with the known data shows that the production indicators of the *K. xylinus* B-12068 culture on several C-substrates are consistent and also surpass the known data on BC productivity for most of the known strains, which mainly showed BC productivity of about 0.5–1.0 g/L per day. Thus, on an optimized medium with glucose, BC production was at the level of 2.0 g/L in 7 days [76]; on lychee extracts—2.53 g/L in two weeks of static fermentation [77]; on fat-like substrates–3.3 g/L in five days of cultivation [78]. When the waste products of the sugar industry or plant hydrolysates were used as components of the nutrient medium, more efficient processes were implemented. Thus, in the work of Tyagi et al. [75], a medium with the addition of molasses preliminarily thermally treated with sulfuric acid and diluted in a ratio (1:4) with a concentration of 45.8 g/L in terms of sugar was used. In this case, the BC yield was 12.6 g/L, and the productivity was 1.8 g/L∙day^−1^. In a culture of *Gluconacetobacter sucrofermentans* B-11267 on a molasses-containing medium with ascorbic acid as an antioxidant, the BC yield reached 2.48 ± 0.12 g/L under static condition and 3.73 ± 0.18 g/L under the dynamic condition in three days of cultivation [79]. The use of enzymatic hydrolysates of miscanthus for growing a symbiotic culture of *Medusomyces gisevii* Sa-12 increased the yield of BC by 20%. Moreover, BC samples had a high crystallinity index (88–93%) and an extraordinarily high content of allomorph Iα (99–100%) [80].

### 3.2. Structure and Properties of BC Synthesized by K. xylinus B-12068

The following factors primarily determine the structure, physical and mechanical properties of BC: the condition of producer strain cultivation, the method of process realization, the aeration conditions, and the carbon nutrition [2,16,42,49,81]. 

**Figure 5:**
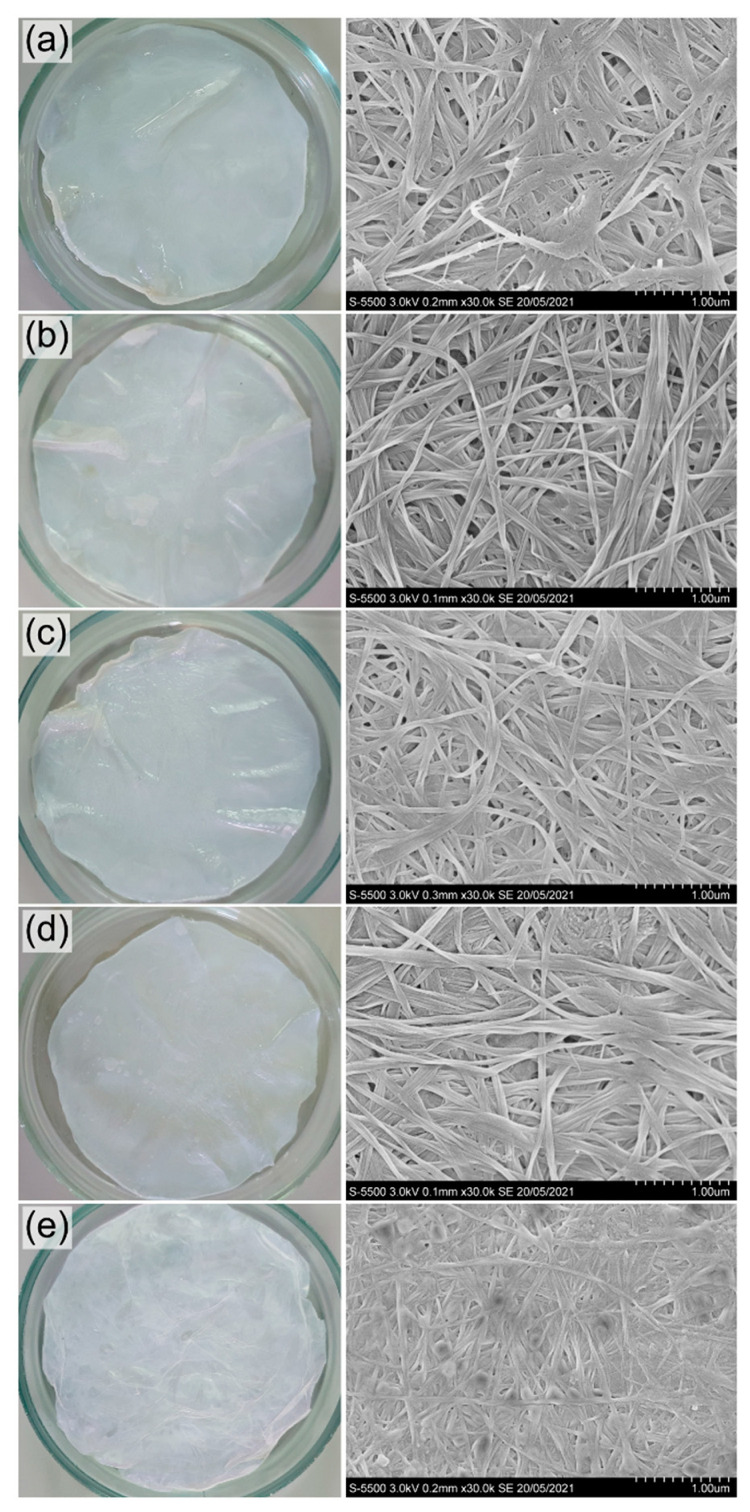
Photo and SEM images of BC films synthesized on various C-substrates: (**a**) glucose; (**b**) glucose + oil; (**c**) molasses; (**d**) glycerol; (**e**) sprat oil. Bar = 1 μm.

The ultrastructure and size of fibrils are critical factors that determine the unique properties of BC. The microstructure of BC films synthesized by *K. xylinus* B-12068 in surface liquid-phase mode on various carbon sources had differences (Figure 5). BC films consisted of undirected fibrils of multiple sizes—cellulose fibers, which formed bundles of different thicknesses.

The average size of fibrils in BC samples synthesized on sugar-containing substrates (glucose and molasses) was close, 62 ± 8 and 50 ± 6 nm, respectively, while the sizes of the formed bundles differed. The largest bundles were formed on glucose medium (275 ± 43 nm), and much smaller bundles were observed on molasses medium (178 ± 27 nm). The thinnest fibrils and bundles characterize the BC sample synthesized on sprat oil, 39 ± 8 and 116 ± 18 nm, respectively. The addition of sunflower oil to glucose did not affect the average fibril size (60 ± 7 nm) but somewhat decreased the size of the bundles to 238 ± 39 nm compared to the glucose samples. The average size of fibrils in the sample synthesized on glycerol was 53 ± 4 nm, with an average size of bundles of 181 ± 27 nm, which was significantly less than the size of fibrils on sugars. The notable differences in the microstructure of BC films synthesized in a surface liquid-phase culture on various C-substrates correspond to the previously obtained results when comparing BC films synthesized by the same strain, cultured in glass flasks under mild stirring on a shaker [57]. During the growth of bacteria on various sugars and a mixture of glucose + ethanol, the samples of BC films also differed in the sizes of microfibrils and their packaging.

We carried out the studying of the physical and chemical properties of the synthesized BC on the films, dried and purified from microorganisms, and obtained in surface culture on a standard HS medium with various carbon sources. 

**Figure 6:**
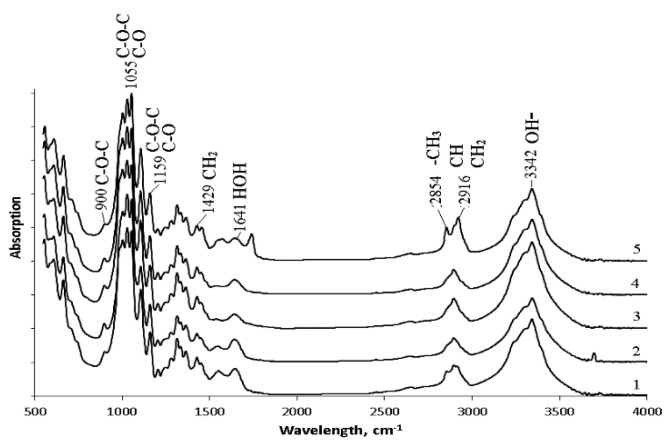
FTIR spectrum data of BC synthesized on various C-substrates: 1—glucose; 2—glucose + oil; 3—molasses; 4—glycerol; 5—sprat oil.

FTIR spectrum of BC is shown in Figure 6. All obtained spectra were identical and had a characteristic set of absorption bands. Stretching vibrations of hydroxyl groups (OH-), which are involved in intermolecular and intramolecular hydrogen bonds, were recorded in the region of 3340–3342 cm^−1^. The absorption bands at 2916 cm^−1^ correspond to asymmetric stretching vibrations of methylene (CH_2_); 2854 cm^−1^ bands relate to symmetric stretching vibrations of the methyl group (CH_3_). All samples showed the presence of bound water (HOH): characteristic absorption bands in the region of 1641 cm^−1^ near 1433 cm^−1^ bands for the asymmetric deformation vibrations of methylene (CH_2_). At wavelengths of 1159 and 1059 cm^−1^, absorption bands characteristic of stretching vibrations of C-O-C and C-O bonds in polysaccharides are recorded. The absorption band at 1159 cm^−1^ corresponds to the stretching vibrations of the C-OH bond of the hydroxyl at the third carbon atom. The absorption band 900 cm^−1^ corresponds to the first carbon atom involved in the formation of the β-glycosidic bond. The obtained results agree with the literature data [82,83].

**Figure 7:**
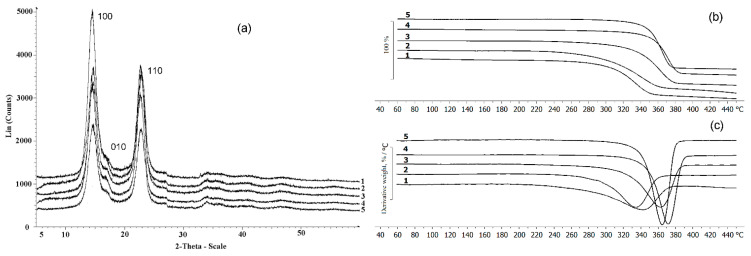
X-Ray (**a**) and TGA (**b**,**c**) data of the BC synthesized on various C-substrates; (**b**) beginning of destruction; (**c**) destruction rate; 1—glucose; 2—glucose + oil; 3—molasses; 4—glycerol; 5—sprat oil.

The bands at 1433 cm^−1^ (CH_2_), 1159 cm^−1^ (C-O-C), and 900 cm^−1^ (group C_1_) can be used to study the crystallinity of BC. An absorption band at 1433 cm^−1^ is a crystallinity one, 900 cm^−1^ is an amorphous band. An increase in the intensity of the absorption band at 1433 cm^−1^ indicates an increase in the degree of crystallinity (Cx). For the studied samples, the highest intensity of the absorption band at 1433 cm^−1^ was recorded for films obtained on glucose + oil and molasses, and the lowest one was on glycerol. The obtained data are confirmed by the results of X-Ray spectroscopy (Figure 7a). The highest degree of crystallinity was recorded for samples produced on glucose + oil and molasses (77%). The lowest degree was for samples on glycerol, and samples on glucose and sprat oil had the same Cx (63%).

In addition to the intensity of these bands, their displacement is also essential. The spectral region 1162 cm^−1^ corresponds to cellulose C-O-C bridges. In crystalline cellulose, this band is located at 1163 cm^−1^, while for amorphous cellulose, it is located at 1156 cm^−1^ [82]. Microbiological synthesis of BC mainly produces long fibers (cellulose I) capable of crystallizing and short fibers (cellulose II), which cannot form crystals due to a lack of fiber length. All samples BC are characterized by an absorption band at a wavelength of 1159–1160 cm^−1^. The 1430 cm^−1^ band is characteristic of crystalline cellulose type I. If the cellulose fiber has a significant content of cellulose type II, this band shifts to 1420 cm^−1^, and the amount of cellulose I decrease [83]. Thus, the set of characteristic bands shows that the synthesized BC samples are mainly represented by cellulose I. Note that bacterial nanocellulose has distinctive properties that are superior to those of plant cellulose. These properties of bacterial cellulose include a high degree of crystallinity and a high content of allomorph Iα in the samples. It is the predominant content of allomorph Iα that allows the identification of the microbial origin of cellulose.

The results of X-Ray analysis and thermal characteristics of the BC are shown in Figure 7. The presence of three characteristic peaks characterizes all BC samples: in the ranges 14.60°, 16.82°, and 22.78°, which corresponds to the main planes of the cellulose crystal (100; 010; 110), and this is consistent with the literature data [84]. As noted above, the type of C-substrate influenced the degree of crystallinity, which varied in the studied samples from 55 to 77%. It was previously also shown [57] that BC samples synthesized by the *K. xylinus* B-12068 strain on various sugars differ in the degree of crystallinity. For example, the BC sample obtained on HS medium with galactose Cx was 45%; on a medium with glucose and sucrose—63 and 68%.

The thermal characteristics of BC synthesized on different C-substrates also differed. The lowest thermal stability was observed in samples obtained on glucose substrate and the mixture of glucose and oil; the temperature of the onset of degradation was 306 and 298 °C, respectively. The highest thermal stability was in BC samples obtained on glycerol and sprat oil when the degradation temperature was 355 °C and 348 °C, respectively. Weight loss of BC sample produced on molasses started at 337 °C. Differential weight loss curves show that the samples had different degradation rates. The BC films synthesized on glucose and mixed substrate were characterized by a lower rate of decomposition, as well as the lowest degradation temperature. The maximum decomposition rate was noted in these samples at 346 and 333 °C, respectively.

The highest degradation rates, expressed as a narrower and elongated peak of the derivative indicating that the decomposition of the sample occurs in shorter temperature and time intervals, were observed in samples produced on glycerol and sprat oil. The maxima of the degradation rates of these samples were recorded at 373 °C and 364 °C, respectively.

Thermal decomposition of BC, as shown in several studies, is determined by specific structural parameters such as molecular weight, degree of crystallinity, and BC fiber alignment [85]. Different conditions of their synthesis cause some differences in thermal stability between pristine BC samples. As the degradation region has no pronounced peaks, it seems reasonable to speak of the decomposition onset temperature (T_dec_. onset). The highest thermal stability was described for BC samples synthesized on the medium with galactose (T_dec_. onset = 284 °C), and the lowest one was described for BC synthesized on sucrose (T_dec_. onset = 220 °C) [57]. A study by Mohammadkazemi et al. [63] showed that the start of thermal decomposition of BC could occur between 200 and 250 °C. Still, more noticeable decomposition, with the samples losing 70–80% of their weight, was observed at 360–390 °C. It is consistent with the data reported by other authors [79], showing the weight loss of the BC sample during thermal decomposition at 300 °C and higher rates of this process at 350–370 °C.

The results of studying the mechanical and surface properties of BC films are presented in Table 3. It is known that the mechanical properties of BC are primarily determined by the BC producer used, conditions of synthesis, fibril thickness, and method of drying of the films. The tensile strength of air-dried BC films varies between 129 and 198 MPa [86], while the tensile strength of freeze-dried ones is an order of magnitude lower (8–14 MPa) [15,29]. Earlier, it was shown that the moisture content of the samples has a strong effect on the mechanical strength of BC [87].

Mechanical parameters of BC films synthesized *K. xylinus* B-12068 did not depend on the type of C-substrate and were similar for all studied samples. The parameters were significantly different depending on the moisture content of the films. So, samples taken out after the fermentation and rinsed to remove the medium and bacterial cells, with a moisture content of over 90%, were as follows: Young’s modulus 10.26 ± 0.35 MPa, tensile strength 0.75 ± 0.34 MPa, and elongation at break 5.49 ± 1.21%. The dry films, with low moisture content (50–55%), only differed from the wet ones in their Young’s modulus, four times higher. BC films, regardless of moisture, had a contact angle below 50°; at the same time, the value for the native raw films was below 41°.

### 3.3. Preparation and Characterization of BC Composites with Nanosilver and Antimicrobial Drugs 

To impart additional properties to BC, such as antibacterial activity, improvement of cell adhesion, and proliferation, BC-based composites with various materials are designed [37,38]. Nanoscale composites are considered especially promising. It is believed that the most acceptable form of materials for restoring tissue defects are BC composites with nanoscale materials, including nanoparticles of gold, silver, cadmium, and oxides of various metals (copper, titanium, zinc, etc.) [22,88,89,90]. The possibility of obtaining BC composites with metal nanoparticles is widely discussed in the literature. Various methods are described for the in-situ production of nanoparticles of silver, copper, or other metals in a cellulose matrix and cotton cloth using various reducing agents and a simpler and more environmentally friendly hydrothermal method. To obtain composites of silver nanoparticles and BC, we used different reducing agents and reactions: polydopamine in the Tollens reaction, sodium citrate dihydrate by the Keri Lee method, borohydride reduction of silver, etc. [40,88,91,92]. The hydrothermal method is a fairly simple, effective, and environmentally friendly method that makes it possible to obtain nanosilver without any additional reagents, in which cellulose itself acts as a reducing agent [58].

**Figure 8:**
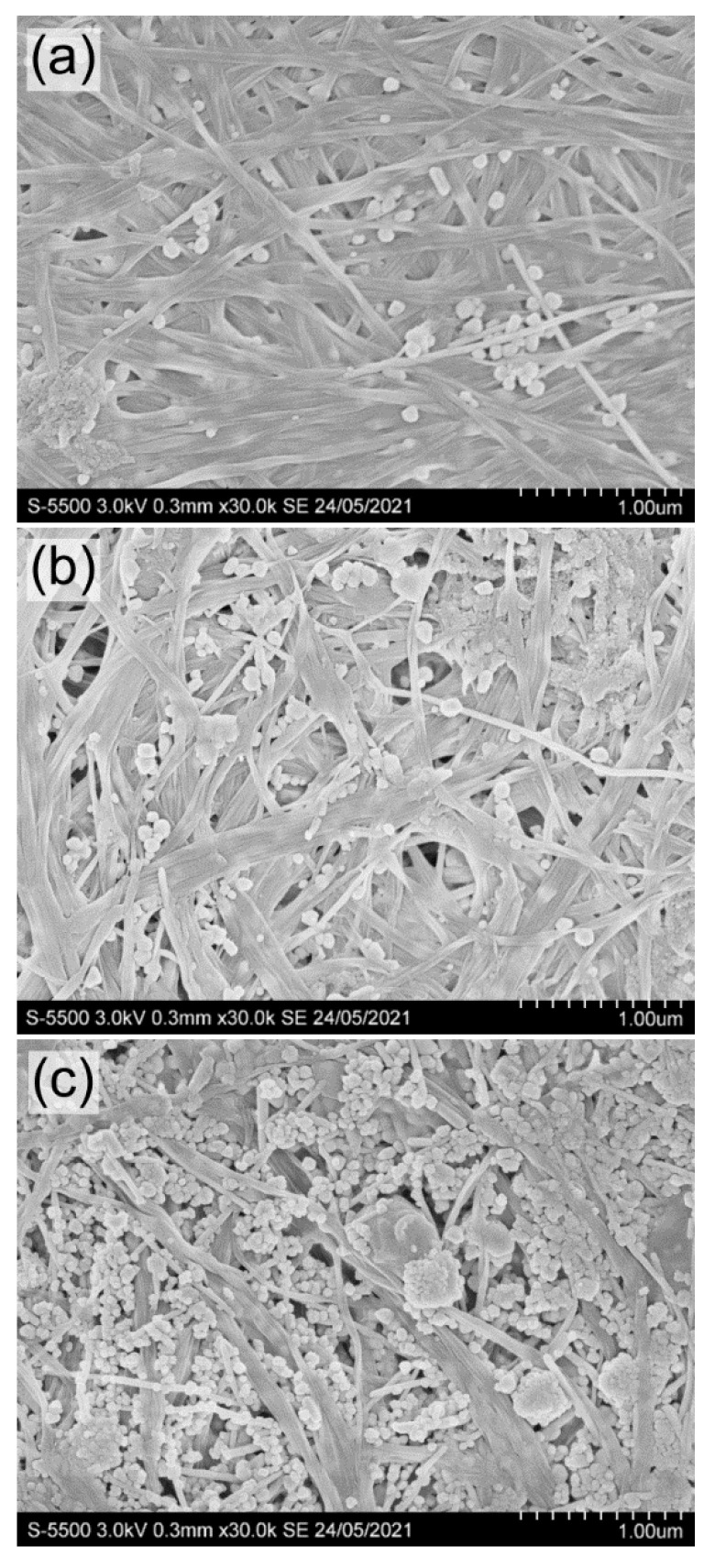
SEM images of BC/AgNP films with different Ag content in the composite: (**a**) 0.06; (**b**) 0.21; (**c**) 0.46 mg/cm^2^ (obtained at a concentration of AgNO_3_ in solution, respectively, 0.01; 0.05; 0.10 g/L). Bar = 1 μm.

SEM images of BC composites with nanosilver BC/AgNPs obtained by the hydrothermal method are shown in Figure 8. 

The presence of silver in BC films was confirmed by elemental analysis performed using scanning electron microscopy equipped with an X-ray spectral analysis system. The amount of nanosilver included in the BC films increased with an increase in the AgNO_3_ concentration in the solution in the studied range (from 0.01 to 0.10 g/L). Quantitative data on the content of nanosilver and the elemental composition of the BC/AgNPs composite films are presented in Table 4.

The concentration of AgNO_3_ in solution influenced the amount of nanosilver in composite BC/AgNP films and the size of Ag particles. At AgNO_3_ concentrations of 0.01 and 0.05 g/L, the particle size varied in the range of 32–76 nm. At the highest concentration of silver nitrate (0.10 g/L), the particles were larger (53–84 nm). The largest agglomerate sizes (from 171 to 396 nm) are recorded at the highest (0.46 mg/cm^2^) nanosilver content in BC/AgNP films. Silver concentration in BC films and silver nanoparticle size obtained in this study are comparable with the data [58] (the size of silver particles of 14–22 nm, silver content reaching 2.3% *w*/*w*) and [93] (the size of particles of between 20 and 100 nm). When silver nanoparticles were produced using reducing agents in the reaction medium, researchers [91,94] noted different sizes of Ag nanoparticles (8–10 nm). 

**Figure 9:**
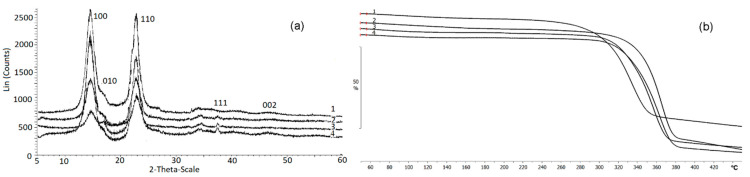
X-Ray (**a**) and TGA (**b**) data of BC and BC/AgNp composites: (1)—pristine BC; (2–4)—BC/AgNp composites obtained at different initial concentration of AgNO_3_ in solution, respectively, 0.01; 0.05; 0.10 g/L.

The inclusion of nanosilver in the BC composites influenced the physicochemical properties, the degree of crystallinity and temperature indicators (Figure 9). The presence of nanosilver in the composites is confirmed by the strong reflexes with coordinates 2θ = 37.72° and 43.89° in radiogram 2, 3 and 4 (Figure 9a). Their positions are consistent with reflexes (111) and (002) of crystalline silver at a cell parameter of 4.126 Å.

With an increase in the content of silver nanoparticles in the composite to 0.21 and 0.46 mg/cm^2^, the degree of crystallinity slightly decreased—to 63 and 58%, respectively. Other authors reported similar data. The C_x_ of BC films/silver varied considerably, between 46.7 and 91.62 [63,95]. Comparable C_x_ values were obtained for the composites with nanosilver synthesized in the presence of NaBH_4_ as a reductant [96]. 

The composites produced with different concentrations of AgNO_3_ in the reaction medium also showed higher thermal stability than the BC (Figure 9b). The thermal degradation temperature (T_degr_) of pristine BC was 306 °C. In all composite samples, the regions of onset of thermal decomposition were shifted to the right relative to BC (330–343 °C). Thus, the incorporation of silver nanoparticles into cellulose films increased the thermal stability of BC. Other authors reported a similar enhancement of thermal stability of BC composites with nanosilver [95].

Properties of BC composites are listed in Table 3. The surfaces of the BC/AgNPs composites are more hydrophobic than the original BC. The values of the contact angle ranged from 50 to 69°. The introduction of nanosilver into the BC reinforced the mechanical strength, increasing the values of Young’s modulus from 99.3 ± 5.2 to 233.0 ± 5.2 MPa, and elongation at break from 7.7 ± 2.5 to 11.7 ± 1.6%. Several publications report that physical and mechanical parameters are determined by the orientation of fibers in BC films, but the data are contradictory. In a study [97], parameters of mechanical strength of BC are considerably higher than the values reported by [98] but comparable to those obtained in the present study. The authors of one more study [99] recorded the ultimate strength of the BC/AgNPs composite at a level of 108 MPa, which was significantly higher than the tensile strength of pristine BC (59 MPa). Thus, in most of the studies, loading of nanosilver into BC led to enhancement of mechanical strength of the films.

The second group of BC composites included antibacterial drugs with different mechanisms of action. BC composites with chlorhexidine, baneocin, cefotaxime, and doripenem were obtained by exposing cellulose films in solutions of these compounds. In contrast to silver nanoparticles, antibiotics, impregnated into BC, did not significantly change the degree of crystallinity and temperature properties of composite films, but affected their surface properties (Table 3). BC composites with antibiotics, like BC/AgNPs, had more hydrophobic surfaces than pristine BC films; their dispersive components and surface energies were also higher. 

The surface of BC composites with antimicrobial agents is more hydrophobic, with a contact angle of about 70°, which exceeds the values for BC/AgNPs composites and even more significantly exceeds the values for the original BC films (Table 3). Mechanical properties of BC composites with antimicrobials were generally lower than those of BC/AgNPs composites; the Young’s modulus was 2–3 times lower, with some superiority of the elasticity index, characterized by the elongation at break. Young’s modulus of BC composite with chlorhexidine was 63.2 ± 1.8 MPa, with baneocin 58.9 ± 6.9 MPa, with cefotaxime and doripenem 44.6 ± 4.2 and 45.5 ± 8.1 MPa, respectively. On the contrary, the elasticity of BC composites with antimicrobials was (elongation at break, %) in general in all samples higher than in BC/AgNPs composites and the original BC. Information on the preparation and use of composites of BC with antibiotics is less representative [79,93,100,101,102].

### 3.4. Antibacterial Properties of BC Composites 

**Figure 10:**
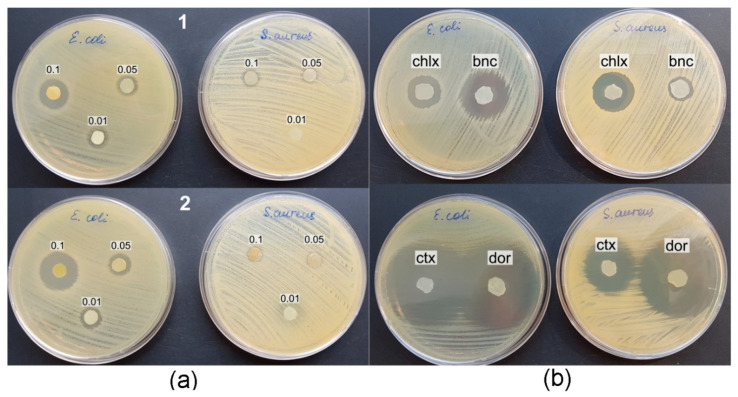
Antibacterial activity of BC composites against reference strains *E. coli* and *S. aureus*; (**a**) BC/AgNPs composites: 1—boiling BC in silver nitrate solution, 2—immersion of BC in silver nitrate solution for 9 days; (**b**) BC/antibiotic composites: *chlx*—BC/chlorhexidine, *bnc*—BC/baneocin, *ctx*—BC/cefotaxime, *dor*—BC/doripenem.

The study of the antibacterial properties of the obtained BC composites was carried out in the cultures of the reference strains *Escherichia coli* ATCC 25922 and *Staphylococcus aureus* ATCC 25923, using the disk-diffusion method in 20 mL agar on Petri dishes. When testing BC/AgNP composites, pronounced zones of growth inhibition were manifested only in relation to *E. coli*, while *S. aureus* was weakly sensitive to the action of silver nanoparticles (Figure 10, Table 5). 

The concentration of the initial solutions of silver nitrate and the amount of nanosilver included in BC influenced the antimicrobial activity of the composite films. The largest diameter of the inhibition zone for *S. aureus* growth was observed using the composites obtained with the highest concentration of the starting AgNO_3_ solution (0.10 g/L). A decrease in the AgNO_3_ concentration by a factor of 2 and 10 (to 0.05 g/L and 0.01 g/L) reduced the biological activity of the composites. 

The diameter of the zones of bacterial growth inhibition by composite films obtained with the thermal incorporation of Ag into BC films from silver nitrate solution was 1.1–1.3 times smaller than that of films obtained by exposing BC in an AgNO_3_ solution at room temperature for 9 days. Apparently, for the process of silver reduction and the inclusion of silver nanoparticles in BC films, a more significant factor is the concentration of silver salt in the solution and the duration of exposure of the BC film, rather than the temperature of the reaction medium. The combination of the highest concentration of AgNO_3_ in solution and long-term exposure to BC films increased the antibacterial activity of the BC/AgNP composite. The diameter of the no-growth zone of *E. coli* was 21.1 ± 1.2 mm (Figure 10a).

Composite BC/AgNP films obtained by replacing silver nitrate with silver citrate had a weak antimicrobial effect against reference bacterial strains. Pumping of silver citrate solution through BC-film did not promote the appearance of antimicrobial activity in the resulting composite. Only at a high concentration of silver citrate (0.4 g/L) a slight suppression of the growth of *E. coli* with an inhibition zone diameter of about 9.2 ± 0.2 mm was found.

Comparison of the obtained results with the published ones showed that the preparation of BC/AgNP composites by the hydrothermal method and antimicrobial activity was confirmed against *E. coli* and *S. aureus* [103,104], and in combination with chitosan—against *B. cereus, P. aeruginosa,* and *C. albicans* [105]. A study of the mechanism of action of nanosilver on *S. aureus* and *E. coli* showed that under the action of AgNP, oxidative stress occurs in bacterial cells, which leads to DNA damage and accumulation of lipid peroxidation products [106]. It has also been shown that the resistance of a number of bacteria to AgNP may be associated with an increased ability to cause aggregation of nanoparticles and disrupt their stability [107]. Pazos-Ortiz et al. [108] have described the positive dependence of the antimicrobial activity of PCL-AgNPs hybrid nanofibers on the concentration of stock silver nitrate solutions. The authors of this work found a higher sensitivity of Gram-negative bacteria to silver nanoparticles since a thick layer of Gram-positive peptidoglycan limits the permeability of the cell wall for silver nanoparticles. The antimicrobial effect of BC composites with nanosilver is also associated with damage to the membrane structures of bacterial cells and disruption of membrane-dependent processes. Thus, it was shown that under the action of AgNP, the outer membrane in *E. coli* cells is disaggregated, as a result of which membrane permeability and cell integrity are disrupted [109]. This work also showed that the interaction of AgNP with thiol groups of sulfur-containing proteins leads to inactivation of dehydrogenases in the inner membrane of cells, suppression of respiration, and a decrease in the energy balance of cells. AgNP particles can also interact with unsaturated fatty acids, change membrane fluidity, causing cell deformation [110,111].

BC composites with antimicrobial drugs more pronouncedly inhibited the growth of the *E. coli* and *S. aureus* reference strains compared to BC/AgNP (Table 5). Immersion of BC in a solution of the antiseptic chlorhexidine provided a pronounced antibacterial effect of the resulting composite against both strains, while the BC/baneocin composite exhibited a strong inhibitory effect against *E. coli* and a weak one against *S. aureus*. BC composites with broad-spectrum antibiotics, cefotaxime and doripenem, effectively inhibited the growth of test bacterial strains (Figure 10b). The diameters of the no-growth zones were maximal and amounted to 42.9 ± 2.3 and 44.9 ± 2.0 for *E. coli* and *S. aureus*, respectively.

The results are consistent with publications showing that BC composite films with antibiotics have high antibacterial activity against both Gram-negative and Gram-positive bacteria. The inhibition of *S. aureus*, *E. coli*, *B. coagulans*, and *Aspergillus niger* with BC loaded with amoxiclav and fluconazole was demonstrated [100]. BC/poly-N,N-dimethyl-3,4-methylene pyrrolidinium chloride composites with embedded selenium and hydroxyapatite nanocomplexes exhibited antibacterial properties, inhibiting the growth of *E*. *coli* [79]. Positive examples of BC inhibition by antibiotics of wound infection of representatives of *Staphylococcus aureus* and *Bacillus subtilis* are described [101]. BC films loaded with tetracycline hydrochloride, BCTCH, exhibited high antibacterial activity [102]. Composite film BC/fusidic acid had an antimicrobial effect against *S. aureus* [112], the combination of BC with tetracycline hydrochloride effectively inhibited the growth of *E. coli*, *S. aureus*, and *B. subtilis* [93]; composite nanofibers of bacterial cellulose with gentamicin also showed high antibacterial activity against *E. coli* and *S. aureus* [113].

## 4. Conclusions

The synthesis of bacterial cellulose (BC) by the strain of acetic acid bacteria *Komagataeibacter xylinus* B-12068 on various C-substrates, including waste, in submerged stirred cultures and static surface conditions, was studied. The strain can assimilate various C-substrates and synthesize BC on glycerin, glucose, beet molasses, sprat oil, and a mixture of glucose with sunflower oil. We revealed differences in the yield of BC with growth in submerged aerated culture and surface static mode. In stirred culture, the bacterial reproduction was more active, and this mode was suitable for obtaining an active inoculum. The most productive process was obtained during inoculum production in submerged culture and subsequent synthesis of large BC films (up to 0.2 m^2^ and more) in a static surface culture. We got the highest productivity of the BC synthesis with the growth of bacteria on molasses and glycerol, 1.20 and 1.45 g/L per day, respectively. 

We obtained the series of different BC composites with silver nanoparticles (BC/AgNPs) and antibacterial drugs (chlorhexidine, baneocin, cefotaxime, and doripenem), and investigated their structure, physicochemical, and mechanical properties. BC composites exerted antibacterial properties against reference bacterial strains *E. coli* and *S. aureus*. The disc-diffusion method showed pronounced antibacterial activity of all BC composites and was more noticeable in composites BC/cefotaxime and BC/doripenem.

## Figures and Tables

**Table 1:** Production characteristics of BC synthesized by *K. xylinus* B-12068 on various substrates.

C-Substrate	Yield BC, g/L	Productivity, g/L·Day^−1^
Glucose	3.22 ± 0.37	0.64
Glucose + sunflower oil	2.21 ± 0.49	0.44
Molasses	3.34 ± 0.30	0.67
Glycerol	3.90 ± 0.30	0.78
Sprat oil	3.23 ± 0.43	0.65

**Table 2:** The influence of cultivation conditions in fermenter Bio-Flo 115 on the production parameters of *K. xylinus* B-12068.

Parameters	Yield of BC, g/L	Productivity, g/L·Day^−1^
Mixer revolutions, rpm		
50	0.53	0.08
250	0.71	0.10
500	2.67	0.38
750	1.40	0.20
Air supply, L/min per L of the medium (at 500 rpm of the mixer)		
0.3	2.92	0.42
0.4	3.33	0.48
0.6	2.15	0.31

**Table 3:** Mechanical and surface properties of BC films and BC composites.

Samples	Physical-Mechanical Properties			Surface Properties		
	Young’s Modulus [MPa]	Tensile Strength [MPa]	Elongation at Break [%]	Water Contact Angle [°]	Dispersive Component [mN/m]	Polar Component [mN/m]
BC native, wet (moisture content 90%)	10.2 ± 1.3	0.7 ± 0.3	5.5 ± 1.2	41.9 ± 3.4	44.8 ± 1.5	20.9 ± 0.7
BC dry (moisture content 50–55%)	47.6 ± 6.3	0.1 ± 0.1	4.4 ± 0.8	48.2 ± 6.7	46.6 ± 0.6	16.8 ± 0.6
BC/AgNPs (moisture content 50–55%)						
BC/AgNPs 0.10 g/L	233.0 ± 5.2	14.9 ± 1.7	9.8 ± 2.6	50.3 ± 4.6	42.6 ± 1.0	27.7 ± 3.6
BC/AgNPs 0.05 g/L	218.0 ± 2.2	13.2 ± 2.1	7.7 ± 2.5	68.8 ± 1.8	46.2 ± 2.9	22.6 ± 0.8
BC/AgNPs 0.01 g/L	99.3 ± 5.2	9.9 ± 1.3	11.7 ± 1.6	69.0 ± 2.7	44.9 ± 1.8	25.1 ± 0.9
BC/drugs (moisture content 50–55%)						
BC/chlorhexidine	63.2 ± 1.8	6.1 ± 1.8	11.7 ± 2.1	70.4 ± 3.1	45.7 ± 3.1	24.7 ± 1.1
BC/baneocin	58.9 ± 6.9	5.3 ± 0.9	11.9 ± 2.7	70.2 ± 6.4	44.3 ± 2.9	25.7 ± 1.2
BC/cefotaxime	44.6 ± 4.2	6.4 ± 0.4	17.6 ± 1.1	68.4 ± 5.1	42.5 ± 2.1	24.2 ± 2.2
BC/doripenem	45.5 ± 8.1	6.0 ± 1.1	16.3 ± 2.5	72.4 ± 6.3	47.3 ± 3.1	23.2 ± 1.1

**Table 4:** The influence of different AgNO_3_ concentration on the elemental composition of BC/AgNPs composites.

**Samples**	**Average Atomic Number (wt.%)**			**Ag Content (mg/cm^2^)**
	**O**	**C**	**Ag**	
Initial BC	60	41	-	
Concentration of AgNO_3_ (g/L)				
0.01	54	46	2	0.06
0.05	56	43	5	0.21
0.10	53	41	10	0.46

**Table 5:** Inhibition of bacteria grow by BC composites.

Samples	Diameter of Inhibition Zones (mm)	
	*E. coli*	*S. aureus*
Boiling BC in AgNO_3_ solution		
0.10 g/L	15.9 ± 0.9	8.7 ± 0.2
0.05 g/L	10.8 ± 0.6	0
0.01 g/L	9.3 ± 0.5	0
Immersion of BC in AgNO_3_ solution for 9 days		
0.10 g/L	21.1 ± 1.2	8.7 ± 0.5
0.05 g/L	12.1 ± 0.7	0
0.01 g/L	11.4 ± 0.7	0
Immersion of BC in 0.05% water solution of drags		
BC + chlorhexidine	19.2 ± 1.1	22.8 ± 1.3
BC + baneocin	26.7 ± 1.5	14.0 ± 0.8
BC + cefotaxime	42.1 ± 2.2	26.6 ± 1.4
BC + doripenem	44.9 ± 2.0	42.9 ± 2.3

## Data Availability

Not applicable.
